# Investigating the *CYP2B6 *rs3745274 and rs3211371 Polymorphisms in Methadone-Responder and Non-Responder Addicts in Iran

**DOI:** 10.52547/ibj.25.3.220

**Published:** 2021-02-06

**Authors:** Sara Sadat Aghabozorg Afjeh, Behzad Boshehri, Safar Hamednia, Asmaolhosna Asmaolhosna, Parisa Mashayekhi, Mir Davood Omrani

**Affiliations:** 1 Department of Medical Genetics, Shahid Beheshti University of Medical Sciences, Tehran, Iran;; 2 Department of Forensic Medicine and Toxicology, Urmia University of Medical Sciences, Urmia, Iran;; 3 Department of Psychiatry, Urmia University of Medical Sciences, Urmia, Iran;; 4 Sara Medical Genetic Laboratory, Tehran, Iran;; 5 Tajrish Research Center, Pasteur Institute of Iran, Tehran, Iran;; 6 Urogenital Stem Cell Research Center, Shahid Beheshti University of Medical Sciences, Tehran, Iran

**Keywords:** Addiction, Biomarker, Methadone, Single-nucleotide polymorphism

## Abstract

**Background:**

Methadone therapy is a major protocol in opioid addiction cases in many health care systems. Population-based studies have shown that in addicted people, the genetic profile affects their response to methadone therapy. Therefore, this study designed to examine the frequency of two SNPs of the *CYP2B6* gene (rs3745274 and rs3211371) in addicted cases in two methadone-responders and methadone non-responders groups.

**Methods:**

A total of 199 opioid-addicted individuals and 117 unaffected control subjects were genotyped for rs3745274 and rs3211371 polymorphisms of the *CYP2B6* gene using the tetra-primer ARMS-PCR.

**Results:**

Results of this study revealed the significant association of rs3745274 GG (*p* < 0.001; OR = 0.027; 95% CI = 0.14-0.49) and GT (*p* < 0.001; OR = 4.04; 95% CI = 2.26-7.21) genotypes with the risk of addiction in methadone-responders. Also, a significant association between rs3745274 GG (*p* < 0.001; OR = 0.28; 95% CI = 0.15-0.51) and GT (*p* < 0.001; OR = 5.1; 95% CI = 2.8-5.28) genotypes and addiction relapse was found in methadone non-responders.

**Conclusion:**

Based on our findings, we can conclude that rs3745274 variant of *CYP2B6* gene could serve as a potential biomarker, to evaluate the prognosis of addicted people fate under treatment with methadone.

## INTRODUCTION

Addictions, including SUDs, are frequently chronic, with a relapsing-remitting course. SUDs not only affect individuals but also impose many economic, cultural, and health burdens on society and consume a substantial portion of the health system resources every year worldwide^[^^1^^]^. According to local reports, there are about 2-4 million substance abusers in Iran^[^^2^^]^. Among all substances, opium and opium residue are the most commonly used drugs in Iran^[^^3^^]^. At present, biological research has a significant role in reducing this social problem by expanding the range of information on addiction's origins and neurobiology and discovering new treatment strategies^[^^4^^]^. This level of study can provide a comprehensive view of the genetic effects, biological drug targets, neurotoxicity, and relevant signaling pathways that may lead to neuronal adaptation processes and subsequent uncontrolled substance use, withdrawal, temptation to consume, and relapse. 

Given the irreparable harm of addiction and the complexities of treatment, finding genetic factors that can predict a person's susceptibility to addiction can reduce the number of addicts and speed up the treatment process for their withdrawal^[^^5^^]^. Twin studies have implied that genetic factors play a significant role in developing opioid addiction^[^^6^^]^. In fact, one of the main questions is why some people are more at risk of becoming addicted and abusing drugs than others. So far, many studies have been conducted to explore the genetic basis of addiction and have identified several candidate genes that their polymorphisms are associated with the development of addiction^[^^4^^,^^7^^-^^11^^]^. Besides, genes coding for metabolic enzymes, especially CYP enzymes, have been reported to be linked with response to methadone treatment in opioid addicts. In fact, liver CYP enzymes, particularly CYP3A4, CYP2B6, and CYP2D6, are responsible for the metabolism of many medications, such as methadone, which undergoes stereo-selective N-demethylation^[^^11^^,^^12^^]^. Also, highly polymorphic *CYP *genes have shown inter-ethnic differences in allele frequencies^[^^13^^]^. 

*CYP2B6 *SNPs have been displayed to be significantly related to higher (S)-methadone plasma levels^[^^14^^-^^16^^]^. Because of the functional similarity between methadone and opioids, genes affecting the methadone metabolism may influence the development or risk of opioid addiction^[^^17^^]^. To further extend previous studies on the relationship between *CYP2B6 *SNPs and the metabolism of methadone, we investigated the potential effects of two polymorphisms of *CYP2B6 *gene on the development of opioid addiction in a well-characterized sample, including two groups of patients. Group A took advantage of methadone with no co-medication, and group B were treated with adjuvant therapy, in addition to methadone, and did not have sufficient response to methadone treatment. 

## MATERIALS AND METHODS

**Sample Collection**

In this study, 99 opioid addicts on methadone maintenance therapy (group A) and 100 opioid addicts who were on adjuvant therapy with methadone, including gabapentin, risperidone, clonazepam, hydroxyzine, and other medications (group B), were selected as patient/case groups. All patients were diagnosed by the psychiatrists and clinical staff of the Ayatollah Taleghani Hospital of Urmia, West Azerbaijan Province, Iran. Besides, 117 healthy participants who were age- and sex-matched with the patient groups were selected with the following inclusion criteria: (1) they had not used any substance (except smoking) and (2) they had no psychiatric or neurological disorders or had not taken any drugs for medical conditions. Venous blood sample (5 mL) collected from participants and genomic DNA was isolated from peripheral blood leukocytes using the salting-out method according to the protocol described before^[^^18^^]^. DNA concentrations were measured using a NanoDrop 2000c Spectrophotometer (Thermo Fisher Scientific; Wilmington, Delaware, USA).

**Tetra-primer ARMS-PCR**

Primers were designed using the Allele ID6 and Oligo7 software following the protocol explained by Ye *et al.*^[^^19^^]^ and were synthesized by Pishgam Co. (Tehran, Iran). Primer sequences are presented in Table 1. Samples were genotyped for the CYP2B6 516G>T (rs3745274) and 1459C>T (rs3211371) SNP using tetra-primer ARMS-PCR. Each PCR reaction was carried out in a total volume of 10 µl containing 30 ng of the extracted DNA, and 1 pmol of each inner and outer primer (Table 1), 200 µM of dNTP, MgCl_2_, and 0.5 U Taq polymerase (Life Technologies, USA). PCR was performed as one cycle of 95 °C for 5 min, followed by 30 cycles of 95 °C for 40 s, 62 °C for 35 s (according to the annealing temperatures for different PCRs shown in Table 1), 72 °C for 30 s, and an additional 10-min extension at 72 °C. PCR products were analyzed by 1.5% agarose gel electrophoresis in Tris/Borate/EDTA 1× buffer.

**Table 1:** Primers used in the tetra-primer ARMS-PCR measurement method

**SNP**	**primers**	**Sequence (5'->3')**	**Length**	**Tm**	**GC (%)**	**Product size **
rs3211371	F inner	GCAAAATACCCCCAACATACCAGAGCC	27	65.94	51.85	for C allele: 182 for T allele: 231
	R inner	CCTTCAGCGGGGCAGGAATCA	21	64.80	61.90	
	F outer	TATGCACCTGCCCTGTGCCCACA	26	68.69	60.87	two outer primers: 356
	R outer	AGGGGAAGGAAGCTGGCTTGTA	22	64.28	57.14	
						
rs3745274	F inner	CTCATGGACCCCACCTTCCTCTTCTAG	27	65.52	55.56	for G allele: 237 for T allele: 288
	R inner	AGCAGATGATGTTGGCGGTAATGAAA	23	63.47	42.31	
	F outer	AGCCTCTCGGTCTGCCCATCTATAAAC	27	65.80	51.85	two outer primers: 479
	R outer	CAAGACAGGTCATCCTTTTCTCGTGTGT	28	65.09	46.43	

*Tm, melting temperature*

**Fig. 1:**
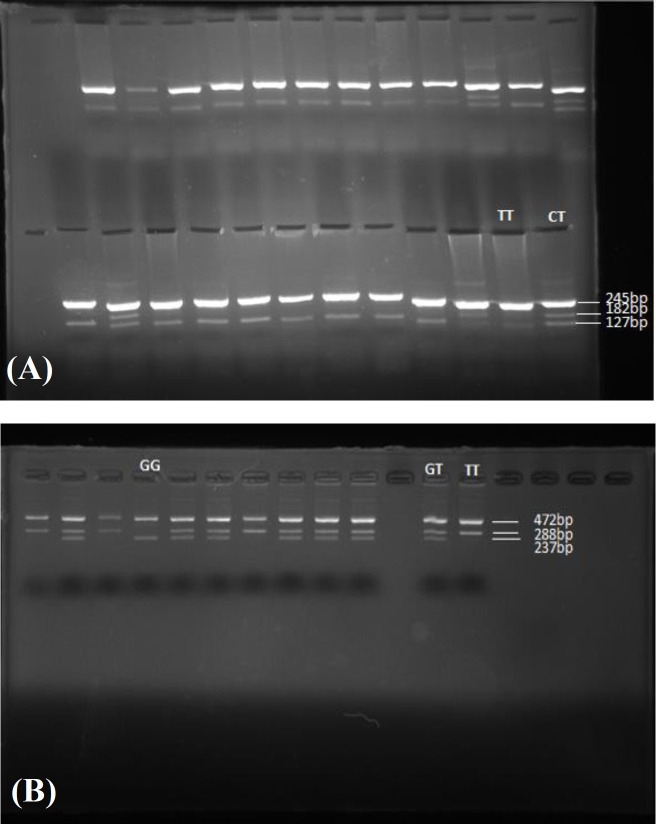
Outer image of (A) rs3211371 and (B) rs3745274 gel electrophoresis

**Statistical analysis**

The Microsoft Excel 2019 and SPSS 24.0 statistical software (SPSS, Chicago, IL) were applied for the statistical analysis of the data of this case-control study. Both patients and control groups were analyzed using χ2 test to determine the fitness to the Hardy–Weinberg equilibrium. The χ2 test was also used for comparing genotype and allelic frequencies between the methadone-dependent subjects and controls. The ORs and 95% CI were calculated for this comparison. In all analyses, *p *values were two-sided and statistically significant if less than 0.05.

**Ethics statement**

The above-mentioned sampling protocols were approved by the Ethics Committee of Shahid Beheshti University of Medical Sciences, Tehran, Iran (ethical code: IR.SBMU.MSP.REC.1398.213). Informed consents were signed by all participants.

## RESULTS

The outer images of genotyping of rs3211371 and rs3745274 are shown in Figure 1. Allele frequencies and genotyping of the both polymorphisms in addicted subjects and unaffected control group were determined using the tetra-primer ARMS-PCR (Tables 2 and 3). As represented in Table 2, in the addicted group, the frequencies of the G and T alleles of rs3745274 polymorphism were 58.5% and 41.5%, while those of C and T alleles were 91.3% and 8.7%, respectively for rs3211371 polymorphism.

Genotype distribution among the addicted people (group A and B) as well as unaffected controls is shown in Table 3. Genotype frequency of the rs3745274 polymorphism in group A in comparison to the control subjects was 20.3% vs. 48.7% for the GG homozygous, 6% vs. 10.2% for the TT homozygous, and 73.7% vs. 41% for heterozygous GT. Analysis showed no significant association between rs3211371 polymorphism and the risk of relapse of addiction. As depicted in Table 4, there was a significant association of rs3745274 GG (χ^2^ = 19.008; *p* < 0.001; OR = 0.027; 95% CI = 0.14-0.49) and GT (χ^2^ = 23.290; *p* < 0.001; OR = 4.04; 95% CI = 2.26-7.21) genotypes with the risk of addiction in methadone-responders in the studied population. Moreover, rs3745274 genotype frequencies in group B compared to the control group were 21% vs. 48.7% for the GG genotype, 1% vs. 10.2% for the TT genotype, and 78% vs. 41% for GT genotype. There was also a significant association of rs3745274 GG (χ^2^ = 17.991; *p* < 0.001; OR = 0.28; 95% CI = 0.15-0.51) and GT (χ^2^ = 30.271; *p* < 0.001; OR = 5.1; 95% CI = 2.8-5.28; Table 4) genotypes with the risk of addiction in methadone non-responders in Iranian population.

**Table 2:** Allele frequencies in the addicted subjects and unaffected controls

**SNP**	**Allele**	**Addicted subject no. (%) **	**Control** **No. (%) **
rs3745274	G T	233 (58.5) 165 (41.5)	162 (69.2) 72 (30.8)
			
rs3211371	C T	336 (91.3) 32 (8.7)	219 (93.5) 15 (6.5)

**Table 3:** Genotype distribution among the addicted cases and unaffected controls

**SNP**	**Genotype**	**Group A** **no. (%)**	**Group B** **no. (%)**	**Controls** **no. (%)**
rs3745274	GG GT TT	20 (20.3) 73 (73.7) 6 (6)	21 (21) 78 (78) 1 (1)	57 (48.7) 48 (41) 12 (10.2)
				
rs3211371	CC CT TT	85 (85.8) 14 (14.2) 0 (0)	73 (73) 16 (16) 1 (1)	103 (88) 13 (11.2) 1 (0.8)

## DISCUSSION

The prevalence of opioid addiction has increased to epidemic levels; however, therapeutic interventions remain limited. Despite the effectiveness of methadone treatment, there is always a risk of relapse^[^^20^^]^. Based on reports, about 46% of addicted patients continue to use opioids during or after the methadone treatment. It is not yet clear how interactions between genes, environment, and drugs can affect the recurrence of addiction^[^^21^^]^. Recently, numerous studies have demonstrated that *CYP2B6* gene is involved in methadone metabolism and clearance and plasma concentrations^[^^22^^-^^24^^]^. It has also been reported that some variations of *CYP2B6* could help to identify subjects at risk for methadone toxicity or relapse of addiction^[^^25^^-^^27^^]^. 

This study aimed to discover the association of rs3745274 and rs3211371 SNPs of the *CYP2B6* gene with opioid addiction relapse. For further understanding the role of these SNPs, samples of addicted patients were divided into two groups based on the response of individuals to methadone treatment. Group A included opioid-addicted patients who remained on methadone maintenance therapy, and group B included opioid-addicted patients who did not show appropriate response to methadone therapy and, therefore, were under adjuvant therapy. The rs3745274 is a missense polymorphism within the *CYP2B6* gene and has been widely reported to be involved in various responses to some medications, including methadone^[^^28^^-^^30^^]^.

Results of this study showed that GG and GT genotypes of rs3745274 are significantly associated with the risk of addiction in both group A and group B of addicted patients. This result highlights the possible role of this variant of *CYP2B6* gene in the effectiveness of medications in opioid-addicted patients. Given the importance of this polymorphism in drug metabolism, it is not unreasonable to expect that it can affect the effectiveness of various substances and cause the recurrence of addiction in patients under treatment. Our results also indicated no significant association between rs3211371 SNP and the risk of opioid addiction. This SNP is a missense polymorphism within the *CYP2B6* gene that has previously been reported in association with methadone metabolism^[^^31^^]^. This lack of association may be due to the small number of samples and the limitations of the study. 

**Table 4:** Genotypic model analysis of the association of rs3745274 polymorphism with groups A and B

**Subjects**	**Genotypes**			**Total**	***p*** ** value** ^*^
	**GG**	**GT**	**TT**		
Group A	20 (20.2%)	73 (73.7%)	6 (6%)	99	<0.001
Control	57 (48.7%)	48 (41. %)	12 (10.3%)	117	<0.001
Total	77	121	18	216	
χ2	19.008	23.290			
OR	0.027	4.04			
95% CI					
Lower bound Upper bound	0.14 0.49	2.26 7.21			
Group B	21 (21%)	78 (78%)	1 (1%)	100	<0.001
Control	57 (48.7%)	48 (41%)	12 (10.2%)	117	<0.001
Total χ2	78 17.991	126 30.271	13	217	
OR	0.28	5.1			
95% CI Lower bound Upper bound	0.15 0.51	2.8 5.28			

^*^Pearson χ2 test

According to the results of this study, rs3745274 variant of *CYP2B6* could be considered as a potential biomarker for evaluating the prognosis of addicted patients fate under treatment with methadone. Additional studies are also necessary to find other relevant variants of *CYP2B6 *gene and understand the underlying mechanism by which the rs3745274 SNP influences the susceptibility to opioid relapse. 
